# The Anticancer Perspective of Tangeretin: A Small Review

**DOI:** 10.3390/molecules30020300

**Published:** 2025-01-13

**Authors:** Yuan Xu, Xi Yan, Junpeng Zhuang, Haijun Hao

**Affiliations:** 1 Department of Organic Chemistry, College of Chemistry, Beijing University of Chemical Technology, Beijing 100029, China; 2022160084@buct.edu.cn (Y.X.); zhuangjp@mail.buct.edu.cn (J.Z.); 2 College of Chemistry, Beijing Normal University, Beijing 100875, China

**Keywords:** flavonoids, tangeretin, biological activity, anticancer substance, chemotherapy, natural compounds

## Abstract

Cancer is an important disease that threatens human life and health. Many natural compounds from plants have been found to have a better inhibitory effect on cancer, and flavonoids are one of them. Tangeretin, a flavonoid, is widely present in a variety of citrus plants and has been shown to have a variety of biological activities that can inhibit tumor cells. Tangeretin can inhibit the growth, proliferation, and metastasis of cancer cells by acting on JAK/STAT (Janus Kinase/signal transducer and activator of transcription) and caspase-3 signal transduction and by regulating the cell cycle of tumor cells. Tangeretin can also work with other chemotherapy drugs, such as cisplatin, to reduce the drug resistance of cancer cells and improve the therapeutic effect of chemotherapy drugs. This review summarizes the effects of tangeretin on various cancers.

## 1. Introduction

Cancer is an important disease that endangers human health. It is the second leading cause of death in the world, and it poses a huge challenge and burden to individuals, families, and even entire societies [1,2]. According to the Global Cancer Incidence and Death Statistics 2022, there were nearly 20 million new cases of cancer in 2022 and nearly 9.7 million deaths from cancer, and there are likely to be more new cases each year in the future [3]. Chemotherapy is an important means of cancer treatment [4], and new compounds have been found to have positive effects on cancer treatment, which may provide us with a new way to treat cancer.

Plants are important sources of natural drugs, and many natural compounds extracted from plants have good therapeutic effects on cancer, such as steroidal saponins [5]. Flavonoids are widely found in plants and are secondary metabolites of plants. They can exist as glycoside ligands or several derivatives of glycosylation, acetylation, methylation, and sulfation [6]. Flavonoids are also commonly found in vegetables and fruits in our daily diet [7,8,9]. Flavonoids are plant-derived polyphenolic compounds with a 15-carbon flavonoid skeleton of C6-C3-C6 with a benzene ring attached to the 2-position of benzopyran [10]. A wide variety of flavonoids have shown good biological activities in the treatment of cancer, such as genistein [11], quercetin [12,13,14], chalcone [15,16,17], and others.

**Figure 1:**
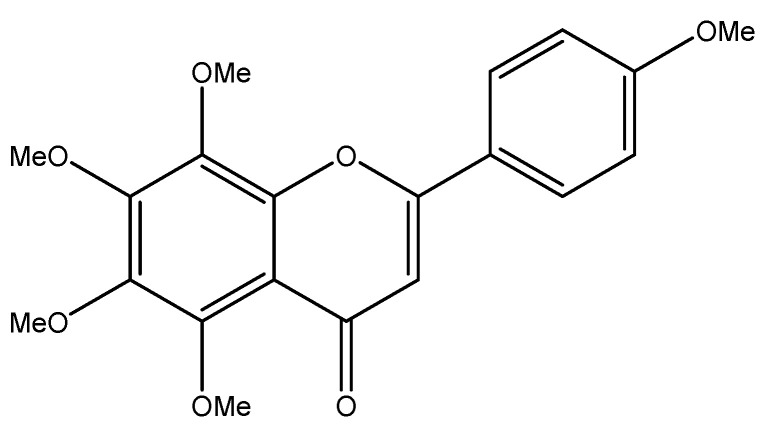
The structure of tangeretin.

Tangeretin (5,6,7,8,4′-pentamethoxy flavonoid) is an important flavonoid, and its structure is shown in Figure 1 [18]. Tangeretin is widely found in citrus plants, and its content can be determined using liquid chromatography and other methods [19]. The content of tangeretin varied in different varieties of citrus plants and in plants produced in different regions. According to reports in the literature, among the 13 kinds of citrus fruits tested, tangeretin was detected in all except three kinds, with the content ranging from 0.28 to 19.20 mg/L [20]. Tangeretin is mainly obtained by extracting it from citrus fruits, especially citrus peels. After a citrus fruit is eaten, the peel is often directly discarded as non-usable waste; therefore, if a large number of low-cost raw materials can be extracted from citrus peels at the same time, the chemical substances in citrus peels can be fully utilized. Traditional extraction methods, which include water extraction, impregnation extraction, and solid-phase microextraction, have a low efficiency and a high cost. It has been reported that the extraction efficiency of liquid carbon dioxide and an ethanol entrainment agent is higher than that of traditional ethanol extraction [21], and it has also been proposed that ultrasonic-assisted extraction could be used to select a more environmentally friendly solvent, reduce the amount of solvent, reduce the extraction time, and improve the effectiveness [22]. Tangeretin has good biological activity; it can play antioxidant, anti-inflammatory, anticancer, and bacterial mutagenesis inhibition roles [23], in addition to others. This article mainly introduces the growth-inhibitory effect of tangeretin on 12 common human tumors. It provides useful information that can guide future researchers and support their work in finding new ways to fight cancer.

According to studies, tangeretin can reduce the proliferation of human tumor cells by affecting the cell cycle and blocking cells in the G1/S phase. At the same time, it has non-toxic and reversible effects on cells at a low concentration and induces death at a high concentration. It may be possible to use tangeretin at a low concentration as an adjuvant drug for chemotherapy. Tangeretin affects cell activity by affecting gene expression. The MAPK (mitogen-activated protein kinase) signaling pathway is an important regulatory pathway for cell cycle regulation and apoptosis, and tangeretin can significantly affect the ERK and p38 pathways and inhibit the phosphorylation of ERK1/2 [24].

## 2. The Anticancer Potential of Tangeretin

### 2.1. Lung Cancer

Maha M. Abdel-Fattah et al. conducted a controlled experiment with male BALB/c mice as the experimental objects. It was found through a histopathological study that, compared with a control group, the number of lung tissue tumors in rats treated with ethyl carbamate significantly increased. Feeding the rats tangeretin in the amount of 200 mg/kg daily for four weeks after the treatment with ethyl carbamate resulted in a significant reduction in the number of tumors compared with the former treatment. At the same time, the authors conducted a progressive biochemical study and found that, compared with the control group, EC (endothelial cell) significantly increased the activity of MPO (myeloperoxidase) and the level of ICAM-1 (intercellular cell adhesion molecule-1), promoted the expression of the p-JAK, JAK, p-STAT-3, and STAT-3 proteins, and significantly increased the immunohistochemical expression of the inflammatory marker NF-ĸB. At the same time, the expression of the apoptosis marker caspase-3 was significantly decreased. Compared with the ethyl carbamate group, the MPO activity and ICAM-1 level in the tangeretin-treated group were significantly decreased; the expression levels of the p-JAK, JAK, p-STAT-3, and STAT-3 proteins were significantly decreased; the expression of NF-ĸB was significantly downregulated. The expression of caspase-3 was significantly increased. Compared with the control group, citrine alone had no effect on MPO or ICAM-1, and there was no significant effect on NF-ĸB or caspase-3 expression. These results indicate that tangeretin alone does little harm to healthy rats at a certain dose, but it can promote a reduction in inflammation and the apoptosis of cancer cells in mice with lung cancer induced by ethyl carbamate. Because the JAK/STAT-3 pathway plays a key role in the development and survival of various cancer cells, abnormal JAK/STAT-3 signaling can lead to tumor metastasis, and tangeretin can reduce the expression of the p-JAK, JAK, p-STAT-3, and STAT-3 proteins while simultaneously inhibiting the growth and survival of cancer cells. According to their study, it can be concluded that, in general, citrulline acts against lung cancer by regulating NF-κB/ICAM-1, JAK/STAT, and caspase-3 signaling [25].

The in vitro treatment of lung cancer cell models with different concentrations (25, 50, and 100 µM) of tangeretin has been reported in the literature. It has been found that tangeretin can significantly inhibit the proliferation of cancer cells, and the inhibitory effect is more obvious at high concentrations. This study showed that tangeretin, when combined with TRAIL (tumor-necrosis-factor-associated apoptosis-inducing ligand), significantly enhanced the anti-lung-cancer effect. The downregulation or deletion of the expression of death receptors DR4 and DR5 is the main mechanism of TRAIL resistance, and tangerine can upregulate the expression of DR4 and DR5 and downregulate the expression of DcR1 and DcR2 (TRAIL antagonistic decoy receptor); therefore, the combination of tangerine and TRAIL can significantly increase the apoptosis of cancer cells. In the expression of TRAIL receptors induced by tangeretin, the upregulated expression of DR4 and DR5 is closely related to the activation of JNK and ERK. The increase in ROS levels observed in this study, together with other studies, suggests that ROS may be involved in the upregulation of DR and ERK/JNK MAPKs. At the same time, it was found in this study that the induction of tangeretin-mediated apoptosis occurred partly through the direct stimulation of apoptosis, and it was partly mediated by CHOP (Cyclophosphamide, Hydroxydaunorubicin, Oncovin, and Prednisone). In addition, CHOP plays a key role in DR5-mediated TRAIL-induced apoptosis. Tangeretin can also promote the apoptosis of lung cancer cells by downregulating the expression of survival proteins and upregulating the expression of pro-apoptotic proteins in a dose-dependent manner [26].

Tangeretin can also be used as an adjunct to reduce lung cancer’s resistance to chemotherapeutic agents. Researchers have reported that ROS (reactive oxygen species) and Nrf2 (nuclear factor erythroid 2-related factor 2) are significantly elevated in drug-resistant human non-small-cell lung cancer (NSCLC), and the expression of Nrf2 indicates that elevated ROS levels and an increased antioxidant capacity are common features of drug-resistant lung cancer cells. Tangeretin overcomes drug resistance by inhibiting Nrf2 and increases the intracellular accumulation of drugs by inhibiting Nrf2/P-gp, thus making lung cancer cells sensitive to chemotherapy drugs. Experiments have shown that, compared with PTX (paclitaxel) alone, the percentage of apoptotic cells increased from 7.6% to 51.0% after a combined treatment with PTX [27].

The design and synthesis of tangeretin derivatives is one of the effective ways to discover better anticancer drug molecules. Some researchers have synthesized the herceptin derivative 5-acetoxy-6,7,8,4′-tetramethoxy-flavonoid (5-AcTMF) and have carried out anticancer studies. They demonstrated that 5-AcTMF effectively inhibited cancer cell proliferation, induced G2/M-phase arrest associated with CDC2 and CDC25c, and increased the apoptotic cells associated with caspase activation. The downregulation of Bcl-2, XIAP, and survivin induces the release of cytochrome c into the cytosol and disrupts the mitochondrial membrane potential. They also found that the 5-AcTMF treatment of CL1-5 activated autophagy by triggering autophagosome formation and increasing the LC3-II levels and LC3 point formation; in addition, 5-AcTMF reduced the phospholipoproteinase 3-kinase/Akt/mTOR signaling pathway. The Akt cDNA transfection overexpression of Akt reduced 5-ACTMF-mediated apoptosis and autophagy, and this supported the induction of apoptosis and autophagy by inhibiting the Akt pathway [28]. Another study modified tangeretin into tangeretin–ZnO quantum dots to investigate their effect on metastatic lung cancer. They used the pH sensitivity of tangeretin–ZnO quantum dots to deliver drugs more accurately and found that they could promote the apoptosis of lung cancer cells and inhibit the proliferation of cancer cells, significantly inhibiting the invasion and migration of metastatic lung cancer cells [29].

### 2.2. Breast Cancer

Breast cancer is a worldwide public health problem and is the most common malignant tumor in women worldwide [30,31,32]. Less than 10 percent of breast cancers can be attributed to inherited genetic mutations. Breast cancer is most often associated with environmental and lifestyle factors, some of which can be changed to reduce the probability of developing breast cancer [33]. Thus, the treatment of breast cancer is also the focus of many researchers.

Previous studies have shown the chemotherapeutic effect of tangeretin on breast cancer. Tangeretin has shown promising results in the treatment of DMBA (7,12-dimethylbenzo (a) anthracene)-induced breast cancer in rats, while tangeretin alone has no effect on a variety of important physiological and biochemical markers in healthy rats. DMBA is a polycyclic aromatic hydrocarbon that produces reactive metabolic intermediates (peroxides and superoxide anion radicals) that induce oxidative stress and lead to carcinogenesis in rats. It was found that, compared with rats treated with DMBA only, the total tumor weight, tumor number, and tumor volume of rats treated with tangeretin were decreased; the levels of the oxidative stress markers NO and LPO were significantly reduced; the levels of enzymatic antioxidants were significantly increased. These results suggest that the antioxidant properties of tangeretin may help mitigate the pathological changes induced by DMBA in breast cancer [34]. Another research team looked at the same question by examining more biochemical markers. They examined the serum levels of animals’—tumor markers alpha-fetoprotein (AFP), carcinoembryonic antigen (CEA), and breast-cancer-specific marker (CA15-3)—and found that tumor-induced rats showed significantly increased levels of these markers compared with control animals. However, these levels were significantly reduced in the oral tangeretin group, which validated the power of tangeretin in the treatment of breast cancer. Additionally, the researchers found that the ER, PR, and Her2/neu were normal in the control group and the group treated with tangeretin alone, while the DMBA-induced rats had higher expression levels of the hormones and their receptors. However, the DMBA-induced rats treated with tangeretin exhibited significantly reduced expression levels of these hormones and their receptors, with the expression being closer to the normal level. This demonstrates tangeretin’s chemotherapeutic ability [35]. For rats induced using DMBA, their liver function was affected. The research results showed that, after the DMBA treatment, the liver marker enzyme activity was increased, and the levels of oxidative stress markers and inflammatory markers were significantly increased, while citrine alleviated these symptoms and the liver damage caused by DMBA [36]. At the same time, other studies have shown that tangeretin can effectively protect the kidneys from DMBA-mediated oxidative damage [37].

Researchers have studied the mechanism of tangeretin in the treatment of breast cancer. It has been reported that tangeretin inhibits the proliferation of tumor cells by downregulating the PCNA (proliferating cell nuclear antigen), COX-2 (cyclooxygenase), and Ki-67 levels. This prevents breast cancer cell division in the G1/S phase through the upregulation of p53/p21. By inhibiting CDK (cyclin-dependent kinase) activity, the expression of cyclin D1 and cyclin E was inhibited. Hesperin also showed anti-metastasis and anti-angiogenesis activities by downregulating the expression of MMPs and VEGF [38]. Another study showed that tangeretin was converted into 4′OH tangeretin by recombining CYP1 and CYP1 expressed in MCF7 and MDA-MB-468 cells, halting the cell cycle of cancer cells in the G1 phase, whereas this transformation was not present in the normal breast cell line MCF10A. At the same time, tangeretin can induce CYP1 enzyme activity and CYP1A1/CYP1B1 protein expression in MCF7 and MDA-MB-468 cells [39]. It has also been reported that tangeretin can regulate the energy metabolism of DMBA-induced breast cancer in rats. The EMP (glycolysis pathway) and TCA (tricarboxylic acid cycle) cycle are important energy metabolism pathways in cells. Glycolysis is carried out in the cytoplasmic matrix, producing less energy but requiring fewer steps; the TCA cycle is carried out in the mitochondria, with many steps, but it produces more energy after the products pass through the respiratory chain. EMP products will enter the TCA for further metabolism in aerobic respiration. The activities of hexokinase, phosphofructokinase, and aldolase in the glycolysis pathway of breast-cancer-affected rats induced using DMBA were significantly increased, and the activities of these enzymes were significantly decreased after a tangeretin treatment. DMBA induced pyruvate dehydrogenase (the enzyme associated with the EMP and TCA), and the activity of TCA cycle enzymes, such as isocitrate dehydrogenase, α-ketoglutarate dehydrogenase, and succinate dehydrogenase, decreased significantly in breast-cancer-affected rats. However, the activity increased after the tangeretin treatment. Glucose-6-phosphate is the synthesis precursor of nucleic acids, phospholipids, and other biological macromolecules in glycolysis. Rapidly proliferating tumor cells have a large synthesis requirement for nucleic acids and other macromolecules, and the high activity of glycolytic enzymes provides the material precursors for tumor cells. At the same time, rapidly proliferating tumor cells have excessive energy requirements. Tangeretin inhibits the proliferation and metastasis of cancer cells [40]. Other researchers have used bioinformatic methods to study the inhibition of PTs and mechanisms in metastatic breast cancer, as well as the important role of the PI3K/Akt pathway and related genes (TP53, PTGS2, NFKB1, and PIK3CA) in the anti-metastasis effect of tangeretin on metastatic breast cancer cells [41].

### 2.3. Prostate Cancer

Prostate cancer is an important cause of disease and death in men, and it has been proven that androgens and androgen receptors play an important role in prostate cancer, which is a highly androgen-dependent disease [42,43,44,45]. It has been reported that the tangeretin treatment of adenocarcinoma PC-3 and LNCa can lead to nuclear contraction, chromosome aggregation, and the formation of apoptotic bodies, which can promote the apoptosis of PC-3 cells, while the toxicity to normal cells is negligible. At the same time, tangeretin can inhibit the migration and invasion of prostate cancer. The same researchers also found that the morphology of PC-3 cells was significantly changed after tangeretin treatment, and further studies have shown that tangeretin can effectively inhibit Akt signaling in PC-3 cells; the Akt signaling pathway plays an important role in the maintenance of the tumor phenotype [46]. In addition, tangeretin (25, 50, and 100 μM) significantly inhibited cancer cell viability and induced DNA fragmentation and apoptosis by increasing the expression of caspase-3 and pro-apoptotic proteins (Bad and Bax) and by decreasing the expression of anti-apoptotic proteins (Bcl-2 and Bcl-xL) [47].

There have also been reports of the synthesis of 5,4′-dimethyltangeretin, which has stronger inhibition on LNCaP cells (GI50 14.6 μM) than PMF1 and is less cytotoxic to normal prostate RWPE-1 cells. 5,4′-Dimethyltangeretin upregulated Bad and Bax, downregulated Bcl-2, and activated caspase-3 and PARP in LNCaP cells, thereby inducing apoptosis. It also inhibited the anchored independent growth of LNCaP cells. It triggered p21 gene expression through the demethylation of the p21 promoter region, and it inhibited DNMT3B and HDACs1, 2, and 4/5/9 protein expression through epigenetic regulation. In addition, 5,4′-dimethyltangeretin decreased isolated LNCaP cancer stem-like cells (CSLCs) with a high CD166 mRNA expression [48].

### 2.4. Bladder Cancer

Bladder cancer is one of the ten most common cancers in the world, and it is an important cause of cancer death [49,50,51]. Four bladder cancer cell lines, 82, BFTC-905, T24, and RT4, were treated with tangeretin, and BFTC-905 was found to be the most sensitive to tangeretin. Tangeretin affected the mitochondrial energy metabolism, which is related to the induction of cell apoptosis. It also induced mitochondrial stress, resulting in the increased expression of the Bad protein and the decreased expression of the Bcl2, Bcl-xL, and p-Bad proteins, resulting in mitochondrial dysfunction and, thus, promoting the apoptosis of bladder cancer cells [52]. The effects of tangeretin on bladder cancer are few and need further study.

### 2.5. Leukemia

Some studies have directly used orange juice to conduct experiments, and the components of orange juice (including tangeretin) inhibited P-glycoprotein-mediated [3H] vincristine effects in human myeloid leukemia (K562/ADM) cells, resulting in the intracellular storage of chemotherapy drugs [53]. Another study used tangeretin to reach a similar conclusion: tangeretin inhibited p-glycoprotein function. It significantly affected the cell cycle but did not induce apoptosis [54].

However, other studies have suggested that tangeretin can promote apoptosis. One study reported that tangeretin inhibited the growth of human promyelocytic leukemia HL-60 cells in vitro, in part by inducing apoptosis, without causing serious side effects to immune cells, and it was found that Ca^2+^ and Mg^2+^ could promote tangeretin’s effects [55]. Tangeretin inhibits proliferation by affecting G2/M without affecting the differentiation of K562 cells. PARP cleavage, a decrease in procaspase-7 and procaspase-3, and a slight decrease in procaspase-9 were observed in the experiment. The pre-cell death was not completely inhibited by the use of total caspase inhibitors, indicating that apoptosis was not completely dependent on caspase. The anti-apoptotic proteins Mcl-1 and Bcl-x were also downregulated. In addition, tangeretin was found to activate UPR in K562 cells. The overall findings indicate that tangeretin’s effect on K562 cells has classic mitochondrial caspase-9-mediated and ER-stress-mediated apoptosis signs [56].

### 2.6. Oral Cancer

Oral cancer is the sixth most common malignancy in the world, and many oral cancer cases are related to the oral habits of betel nut chewing, smoking, and drinking [57]. Although early oral cancer is relatively easy to diagnose, there are still many advanced cases [58,59]. According to reports in the literature, the activity of KB cells can be significantly reduced by tangeretin, and the expression of apoptotic genes and anti-apoptotic genes has been detected. Researchers have found that tangeretin significantly reduces the mRNA levels of the anti-apoptotic genes Bcl-2 and Bcl-xL, increases the mRNA expression level of Bax, and significantly increases the pro-apoptotic activity. Tangeretin can promote the apoptosis of cancer cells by regulating the expression of pro-apoptosis and anti-apoptosis genes [60].

### 2.7. Melanoma

Melanoma is one of the most aggressive and progressive forms of skin cancer. It develops from normal pigment-producing cells called melanocytes [61]. Experiments have proven that tangeretin has anti-proliferation and anti-metastasis activities against melanoma [62,63]. Researchers have studied the relationship between the structure of flavonoids and their anti-melanoma activity. Among the four substances they studied, quercetin, tangeretin, 7,3-dimethyltangeretin, and tangeretin, tangeretin was the most effective drug for inhibiting the growth of the B16F10 and SK-MEL-1 melanoma cell lines. The absence of the flavonoid C-2-C-3 double bond forfeits its effect on both cell lines, whereas tangeretin’s more potent anti-proliferative effect is due to the presence of at least three adjacent methylxyl groups [64]. Other researchers have found that tangeretin can promote the production of melanin in melanoma cells and can induce hyperpigmentation by activating melanin production signaling proteins and initiating sustained ERK2 expression; therefore, it has therapeutic potential for the treatment of melanoma and melanoma-related pigmentation loss [65].

### 2.8. Colorectal Cancer

It has been reported that the inhibitory effect of tangeretin on the proliferation of colorectal cancer is through the induction of cell cycle arrest but not the promotion of cell apoptosis. Researchers have added various concentrations of tangeretin to exponentially growing COLO205 cultures and found that the growth was rapidly inhibited and the G0/G1 cell cycle was stalled. The Rb protein is a negative regulator of G1/S transformation and is inactivated by phosphorylation in the G1 phase, which is necessary for progression from the G1 phase to the S phase, while the Cdks2 and 4 proteins are associated with Rb protein phosphorylation. Researchers have found that a reduction in Rd protein phosphorylation is due to the concentration-dependent inhibition of Cdks2 and 4 protein activity, and this has little relationship with the Cdks2 and 4 levels. The p53 tumor suppressor gene has been shown to be a key regulatory molecule in interpreting external signals that induce cell cycle arrest. Studies have found that tangeretin significantly upregulates the levels of p27 and p21 in a concentration-dependent manner, dependent on the p53 activity, while tangeretin induces p53 protein expression in a dose-dependent manner, which may play an important role in anti-proliferation activity [66]. Another study using the HT-29 cell line came to a similar conclusion, but they also concluded that tangeretin does not kill tumor cells. They found that, after the removal of tangeretin, cancer cells could resume normal proliferation and circulation [67]. It has also been reported that tangeretin can induce GADD45α expression and inhibit the growth of colon cancer cells, and this upregulation is related to the chromatin remodeling of a new putative regulatory element in GADD45α intron 1 [68].

Tangeretin can be used in combination with other drugs to more effectively treat cancer. Researchers have studied the synergistic effect of tangeretin and 5-fluorouracil (5-FU), which can increase the expression of microRNA-21 (miR-21) and activate PI3K/Akt signaling. In cells treated with 5-U, tangeretin inhibited the induction of miR-21 and restored the expression of the target PTEN (phosphatase and tensin homolog deleted on chromosome ten). It also decreased Akt activation and induced autophagy [69]. The synergistic effect of the tangeretin and 5-FU treatment can significantly increase the ROS levels, while the overproduction of endogenous ROS caused by oxidative stress can effectively damage the DNA integrity. Compared with tangeretin and 5-FU treatments alone, the combined tangeretin and 5-FU treatment can effectively trigger DNA damage pathways and inhibit DNA repair systems. At the same time, the combined tangeretin and 5-FU treatment induced apoptosis through the JNK-mediated pathway [70]. In addition to the combined effect of 5-FU and tangeretin, some scholars have studied the combined effect of tangeretin and atorvastatin. They prepared nano-system RGD-ATST/TAGECNPs combined with ATST and TAGE, and they conducted experiments on colon cancer cells and cancer-carrying mouse models. The final results revealed that RGD-ATST/TAGECNPs showed the most significant synergistic therapeutic effect, with no significant toxicity to major organs or tissues [71]. There have been studies in the literature on drug delivery methods, and the use of an emulsion delivery system enhances the anticancer efficacy of tangeretin, which can better inhibit the proliferation of colon cancer cells and significantly reduce the incidence and diversity of tumors [72].

### 2.9. Liver Cancer

The liver is the body’s central metabolic organ, regulating energy and lipid metabolism while also having a powerful immune function. Globally, liver cancer is a common and fatal malignancy [73,74]. Tangerine peel extract (BOPE) can inhibit hepatocellular carcinoma Hep3B cells. The main component of BOPE is tangeretin. Studies have shown that BOPE induces the autophagy of human hepatocellular carcinoma Hep3B cells by promoting the production of ROS and the depletion of ATP, and, at the same time, it can induce ER stress by activating the downstream calcium-ion-related AMPK/TSC2/mTOR and DAPK/Beclin-1 signaling pathways [75]. It has been reported that both tangeretin and its metabolite, 4′-hydroxy-5,6,7, 8-tetramethoxy-flavonoid (4′-OH-TMF), inhibits the cell cycle progression of primary hepatocytes. 4′-OH-TMF is a proliferative signal inhibitor at the level of mTOR, and the inhibitors of the PI3K/Akt/mTOR pathway have been extensively studied in cancer therapy, suggesting that citrulline and its metabolites may play an important role in anti-liver cancer [76].

### 2.10. Gastric Cancer

Some researchers have taken the human gastric cancer cell line AGS as the study object and found that tangeretin induced the apoptosis of AGS cells through exogenous and endogenous signaling pathways. The activation of p53 induces mitochondria-mediated apoptosis by upregulating Bax, which promotes the activation of caspase-9, resulting in the activation of downstream caspase in this process. In addition, Fas/FasL-mediated death receptor pathways may interact with mitochondrial signaling pathways via caspase-8-cleaved Bid [77]. Another study also looked at AGS and found that tangeretin-induced cell death is regulated by the KPCE gene. It is considered that KPCE may be a promising therapeutic marker for gastric cancer [78]. Yue Wang et al. suggested that tangeretin induces cell apoptosis through RARβ. They also conducted in vivo studies and found significant tumor inhibition and a high biosafety [79]. It has also been reported that radiotherapy combined with tangeretin can significantly reduce the tumor size, and tangeretin can enhance the radiosensitivity of gastric cancer cells and almost completely inhibit lung metastasis induced by irradiation [80].

### 2.11. Ovarian Cancer

Ovarian cancer is the deadliest gynecological malignancy worldwide, with a very high mortality rate [81,82]. The emergence of drug resistance makes it more challenging to treat this cancer, and it is very important to seek new drugs to treat cancer or reduce cancer drug resistance [83]. Some researchers have studied the effect of tangeretin on cisplatin-resistant human ovarian cancer cells to increase their cisplatin sensitivity. Their results showed that the combination of tangeretin and low-dose cisplatin induced a significantly higher cytotoxic response in cisplatin-resistant human ovarian cancer cells than a treatment with cisplatin alone for ovarian cancer A2780 cells and that the simultaneous treatment of tangeretin-pretreated cells with cisplatin followed by tangeretin yielded significantly better results than that of cisplatin followed by tangeretin. Tangeretin–cisplatin combination therapy blocks drug-resistant cells in the G2-M phase and enhances cisplatin-induced apoptosis through caspase-dependent mechanisms. The tangeretin–cisplatin combination downregulates the PI3K/Akt pathway, causing cancer cells to become sensitive to cisplatin-induced cell death through apoptosis [84].

### 2.12. Osteosarcoma

Osteosarcoma is a common primary bone cancer that is more common in children and adolescents, and its onset peaks during the adolescent growth spurt [85,86]. Nanoparticles (NPs) have been used for drug delivery and cytotoxic drugs, and in combination with chemotherapeutic drugs, nanomaterials provide an effective delivery tool for chemotherapeutic drugs that can improve the pharmacokinetics and site-specific delivery. Tangeretin and H_2_PtCl_6_·H_2_O have been used to synthesize PtNPs, which have then been combined with DOX to act on U2OS cells. The combination significantly reduced the cell viability and proliferation and changed the cell morphology, and the researchers found a synergistic effect on the cytotoxicity of the combination of U2OS. An increased LDH leakage, the production of reactive oxygen species, and increased levels of malondialdehyde, nitric oxide, and carbonyl proteins also caused mitochondrial dysfunction. In addition, the PtNPs increased the oxidative stress and DNA damage in osteosarcoma [87]. These suggest that the combination may play an effective role in the treatment of osteoma cancer.

**Figure 2:**
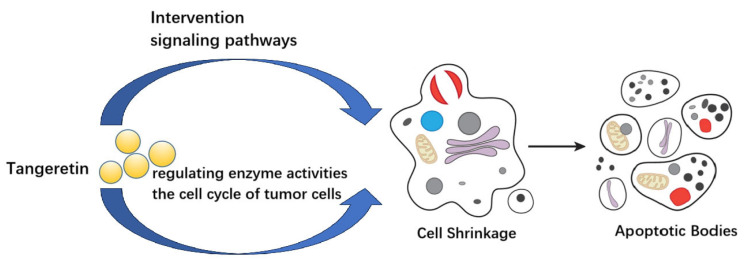
Schematic diagram of the mechanism of action of tangeretin in various cancers.

Figure 2 shows a schematic diagram of the mechanism of action of tangeretin in various cancers based on the content mentioned above.

From Table 1 and Figure 2, it can be seen that tangeretin acts on cells and intervenes in cellular information pathways, the activity expression of various enzymes, and the integrity of the cell cycle. It can cause changes in cell morphology, induces cell shrinkage, and ultimately leads to cell apoptosis. This may be one of the main reasons why tangeretin has the ability to inhibit the growth of various tumors. Tangeretin contains multiple aromatic rings and methoxy groups in its molecular structure, which gives it a better lipophilicity. This property lays the necessary foundation for its ability to penetrate cell membranes and exert anti-tumor activity against various tumors. Tangeretin has certain differences in its anti-tumor targets for different tumors, but its impact on these targets ultimately leads to the inhibition of tumor cell growth, as shown in Figure 2. 

## 3. Other Biological Activities of Tangeretin

Tangeretin has been shown to have many other biological activities, such as the ability to lower blood sugar levels, which may help treat diabetes. The absorption of foodborne glucose is mainly mediated by the sodium/glucose cotransporter 1 (SGLT1). Moderate SGLT1 inhibition can help reduce postprandial blood sugar increases and prevent lifestyle-related diseases. It has been found that tangeretin can inhibit SGLT1 activity in vitro and reduce blood sugar levels in vivo [88]. In addition, other researchers have studied the influence of tangeretin on the liver, the organ targeted by insulin, and found that tangeretin can improve liver insulin sensitivity, both in vitro and in vivo, by inhibiting the MEK-ERK1/2 pathway [89]. According to the above two results, it can be seen that tangeretin has relatively good biological activity in lowering blood sugar, and it could potentially be used as a drug for the treatment of diabetes. In terms of anti-inflammatory and antioxidant properties, tangeretin has also shown considerable biological activity. Some researchers have found that tangeretin has strong anti-inflammatory and antioxidant effects in microglia [90], and tangeretin has neuroprotective and anti-inflammatory effects on I/R (ischemia/reperfusion) injury in rats by inhibiting the inflammatory response [91].

## 4. Conclusions

Tangeretin, a flavonoid with exceptional anti-tumor properties, holds broad promise in oncology. Our comprehensive review elucidates the therapeutic potential of tangeretin across a spectrum of cancers and its synergistic effects when combined with other therapeutic agents. This natural compound exerts its anti-cancer effects by arresting the cell cycle, triggering autophagy, and promoting apoptosis, all while demonstrating negligible toxicity to healthy cells. Our analysis not only underscores tangeretin’s potential as a chemotherapeutic agent or an adjunct to chemotherapy but also highlights its role in cancer prevention, given its prevalence in various diets. Future research endeavors should delve deeper into the anti-tumor mechanisms of tangeretin, and additional clinical trials are imperative to assess its cancer-fighting efficacy in humans. Extracted from readily available orange peels, tangeretin offers a cost-effective and abundant natural resource for its production. We are hopeful that further research on tangeretin will pave the way for more efficacious and safer cancer treatments, ultimately benefiting patients worldwide.

## Figures and Tables

**Table 1:** A brief summary of tangeretin treatments for cancer.

Cancer Type	In Vitro/In Vivo	Experimental Model	Effect	References and Remarks
Lung cancer	In vivo	Male BALB/c mice	Decreased the expression of NF-κB/ICAM-1 and JAK/STAT, and promoted caspase-3 signal transduction	[25]
	In vitro	Human lung cancer cells H1299 and H1975	Promoted DR4 and DR5 expression	[26]
	In vivo and in vitro	Human non-small-cell lung cancer (NSCLC) cell lines NCI-H1819, A549, NCI-H1975, HCC827, and HCT-8 and ABCB1 overexpression-resistant cell line A549/T; female BALB/c nude mouse	Inhibited cellular antioxidant signal Nrf2	[27]
	In vivo and in vitro	NSCLC cell line; BALB/c nude mice without thymus; CL1-5 cells	Induced G2/M stagnation and down-regulated Bcl-2, XIAP, and survivin	[28] (Note: tangeretin derivative 5-AcTMF was used)
	In vitro	NCI-H358 metastatic lung cancer cells	Induced DNA damage of H358 metastatic lung cancer cells; decreased expression of MMP2, MMP9, and VEGF proteins	[29] (using tangeretin–ZnO quantum dots)
Breast cancer	In vivo	Female Sprague Dawley rats (*Rattus norvegicus*)	Decreased the levels of NO, LPO, and CEA and increased the levels of enzymatic antioxidants	[34]
	In vivo	Female Wistar rats	Resulted in anti-proliferation and a low ER, PR, and HER2/neu expression	[35]
	In vivo	Female Wistar rats	Upregulated p53/p21; downregulated PCNA, COX-2, and Ki-67; down-regulated MMPs and VEGF; and inhibited CDK kinase activity	[38]
	In vitro	MCF7 and MDA-MB-468 cells	Caused stasis of cancer cell cycle in G1 phase	[39]
	In vivo	Female Sprague Dawley rats (*Rattus norvegicus*)	Inhibited EMP enzyme activity and enhanced TCA enzyme activity	[40]
Prostate cancer	In vitro	Prostate cancer PC-3 and LNCaP cell lines	Targeted the PI3K/Akt/mTOR signaling pathway	[46]
Bladder cancer	In vitro	BFTC-905 cells	Induced mitochondrial dysfunction	[52]
Leukemia	In vitro	Human promyelocytic leukemia HL-60 cells	Induced apoptosis	[55]
	In vitro	The human erythroleukemia cell line K562	Activated UPR in K562 cells; regulated the expression of Bcl-2 family members in K562 cells; blocked G2/M	[56]
Oral cancer	In vitro	KB cells	Regulated pro-apoptotic and anti-apoptotic genes	[60]
Melanoma	In vitro	SK-MEL5 human melanoma cells	Resulted in anti-proliferation	[62]
	In vitro	B16F10 (highly metastatic subline of mouse melanoma B16)	Resulted in anti-proliferation and anti-metastasis	[63]
Colorectal cancer	In vitro	Cell line COLO 205	Induced G0/G1 cell cycle arrest	[66]
	In vitro	HT-29 (human colorectal adenocarcinoma) cell line	Induced G1 cell cycle arrest	[67]
	In vitro	HCT116 cells	Induced GADD45α expression and anti-proliferation	[68]
	In vitro	Human colorectal carcinoma HCT-116 cells (ATCC) and HCT-15 cells (KCLB)	Induced programmed cell death through JNK-mediated signaling pathway; increased DNA damage and inhibited DNA repair; regulated oxidative stress	[70]
Liver cancer	In vitro	Human liver cancer Hep3B	Induced endoplasmic reticulum-mediated autophagy in human hepatoma cells	[75]
	In vitro	Liver cells isolated from male Wistar-strain rats	Regulated cell cycle progression	[76]
Gastric cancer	In vitro	Human gastric cancer cell line AGS	Caused exogenous and endogenous signaling pathways to induce apoptosis of AGS cells	[77]
	In vitro	Human AGS gastric cancer cell line	Induced cell death	[78]
	In vitro and in vivo	Gastric cancer cell lines AGS, BGC-823, and SGC-7901; BALB/c nude mice (5–6 weeks of age)	Up-regulated RARβ-induced apoptosis	[79]
Ovarian cancer	In vitro	Human ovarian cancer A2780 cells and their homologous cisplatin-resistant A2780/CP70 and cisplatin-sensitive and cisplatin-resistant human ovarian cancer cell lines 2008 and 2008/C13	Downregulated phosphoinositol 3-kinase/Akt signaling pathway	[84]
Osteosarcoma	In vitro	U2OS cells	Resulted in oxidative stress and DNA damage; damaged the integrity of the membrane; increased the levels of NO and carbonyl protein; led to mitochondrial dysfunction; changed the cell morphology	[87]
